# Quantitative myocardial perfusion imaging across PET, SPECT, CMR, and CT

**DOI:** 10.3389/fradi.2026.1760241

**Published:** 2026-03-17

**Authors:** Yoko Kato, Omar Chehab, Bharath Ambale-Venkatesh, Joao A. C. Lima

**Affiliations:** 1 Division of Cardiology, Johns Hopkins University, Baltimore, MD, United States; 2 Department of Radiology, Johns Hopkins University, Baltimore, MD, United States

**Keywords:** absolute myocardial blood flow, cardiac magnetic resonance (CMR), computed tomography (CT), myocardial perfusion imaging (MPI), pixel-wise quantification, positron emission tomography (PET), quantitative, single-photon emission computed tomography (SPECT)

## Abstract

Recent clinical trials demonstrating the prognostic value of coronary computed tomography angiography (CCTA), and the limited incremental prognostic benefit of ischemia-guided revascularization over optimal medical therapy, have shifted focus away from functional testing. Nevertheless, myocardial perfusion assessment remains essential in patients with known or suspected coronary artery disease (CAD), microvascular dysfunction, ischemia with normal coronary arteries (INOCA), coronary anomalies, and cardiomyopathies. Advances in positron emission tomography (PET), cardiovascular magnetic resonance (CMR), and computed tomography (CT) now enable absolute quantification of myocardial blood flow (MBF), providing improved diagnostic and prognostic performance and underscoring the need to reappraise myocardial perfusion imaging. This mini-review summarizes key physiological principles, contemporary acquisition and post-processing strategies, and the clinical relevance of myocardial perfusion imaging (MPI) across PET, Single Photon Emission Computed Tomography (SPECT), CMR, and CT, and discusses future perspectives in quantitative MPI.

## 1 Introduction: clinical indication for stress perfusion assessment

The SCOT-HEART trial (1) in 2018 highlighted the prognostic value of anatomical assessment with coronary computed tomography angiography (CCTA), shifting focus away from functional testing. Similarly, the COURAGE (2007) (2, 3) and ISCHEMIA (2020) (4) trials showed no reduction in coronary events with ischemia-guided revascularization compared with optimal medical therapy. Reflecting these findings, the 2021 American Heart Association chest pain guideline recommends CCTA as the first-line diagnostic test for intermediate-high risk patients with stable chest pain and no prior coronary artery disease (CAD) (5).

Nevertheless, myocardial perfusion assessment remains indispensable in known or suspected CAD, microvascular dysfunction, ischemia with normal coronary arteries (INOCA), coronary anomalies, and cardiomyopathies. The limitations of earlier perfusion-guided revascularization studies such as exercise stress testing, binary interpretations, and outdated protocols, underscore the need to reappraise myocardial perfusion imaging (MPI) (6). Modern positron emission tomography (PET), cardiovascular magnetic resonance (CMR), and computed tomography (CT) techniques now enable absolute myocardial blood flow (MBF) quantification, offering improved diagnostic and prognostic performance (6, 7).

This mini-review summarizes key physiological principles, contemporary acquisition and post-processing strategies, and the clinical relevance of PET, Single Photon Emission Computed Tomography (SPECT), CMR, and CT perfusion imaging, together with a summary table comparing key technical and workflow characteristics of quantitative MPI modalities (Table 1). We further discuss future perspectives in quantitative MPI.

**Table 1:** Comparisons of quantitative MPI modalities.

Characteristics	PET	SPECT	CMR	CT
Procedure time	20–45 min (19)	2.5–4 h (one-day protocols), up to two-day protocols (19)	30–60 min (7)	15–30 min (42)
Radiation Exposure	[^15^O]H_2_O: ∼0.4 mSv; [^13^N]NH_3_: ∼1 mSv; ^82^Rb: ∼0.7 mSv; [^18^F]Flurpiridaz: ∼4.6 mSv (6)	5–12 mSv (19, 21)	No radiation	Dynamic CT perfusion: 5.3 to 13.1mSv (42).
Spatial Resolution	4–6 mm, tracer-dependent (19) [^82^Rb has relatively low spatial resolution. (12)]	10–15 mm [primarily determined by the collimator physics; CZT cameras have a twofold increase in image resolution compared with conventional SPECT cameras (21)]	Minimum spatial coverage is three slices in short-axis orientation, 2.5 × 2.5mm^2^ in-plane, with a maximum slice thickness of 10 mm. (7)	Highest among all modalities, with sub-millimeter spatial resolution (42)
Temporal Resolution	PET detectors count events continuously; for dynamic PET, list-mode data are commonly binned into ∼5-sec frames (62).	CZT SPECT detectors count events continuously; for dynamic SPECT, data are typically binned into 3–10 s frames (30).	High temporal resolution (<120 ms target readout temporal acquisition window), with an effective temporal resolution of one RR interval to ensure a consistent myocardial phase across the dynamic series (7).	The minimum gantry rotation time ranges from 250 to 400 ms, yielding an intrinsic temporal resolution of ∼125–200 ms for single-source CT, while dual-source CT achieves ∼65 ms temporal resolution. Dynamic acquisitions are typically repeated at 1–3 s intervals (42).
Renal Safety/Contrast Considerations	Generally safe in patients with reduced renal function	Generally safe in patients with reduced renal function	Macrocyclic gadolinium agents may be used with caution in patients with severe renal impairment (38) (63).	Iodinated contrast is not recommended in patients with significantly reduced renal function. (64)
Availability	Mostly available in academic or high-volume centers. Requires a PET scanner (often hybrid PET/CT) and an on-site cyclotron for short-lived tracers.	Widely available. Most hospitals have SPECT cameras; CZT cameras are less common but increasingly adopted.	Moderate availability. Requires 1.5 T or 3 T MRI with cardiac capability and trained staff.	Moderate to high availability. Requires a multi-detector CT with perfusion capability (64 + slices or dual-source), which is common in many hospitals. Expertise for dynamic MPI may be limited.
Cost	Radiotracers are expensive, and scanner costs are the highest among all modalities.	Radiotracers are less expensive than PET tracers, and scanner costs are moderate.	Per-exam costs for gadolinium-based contrast agents are comparable to or lower than SPECT tracers; scanner costs are high.	Per-exam costs for iodinated contrast agents are the lowest among the modalities; scanner costs are high.
Key Features	Gold standard for quantitative myocardial perfusion and absolute blood flow measurement; emerging prognostic evidence; limited by high cost and specialized infrastructure.	Widely available and prognostically validated for relative perfusion; quantitative MBF measurement is possible with CZT cameras but less established and lower in accuracy compared with PET.	Excellent spatial and temporal fidelity without radiation; allows multiparametric assessment (perfusion, function, tissue characterization); sensitive to motion and operator-dependent.	Combines high-resolution perfusion imaging with coronary anatomy in a single study; rapid acquisition; limited by radiation and contrast nephrotoxicity.

MPI, myocardial perfusion imaging; PET, positron emission tomography; SPECT, single-photon emission computed tomography; CMR, cardiovascular magnetic resonance; CT, computed tomography; MBF, myocardial blood flow; CZT, cadmium zinc telluride; MRI, magnetic resonance imaging.

## 2 Principles of myocardial perfusion modeling and the essential role of arterial input function (AIF)

**Figure 1:**
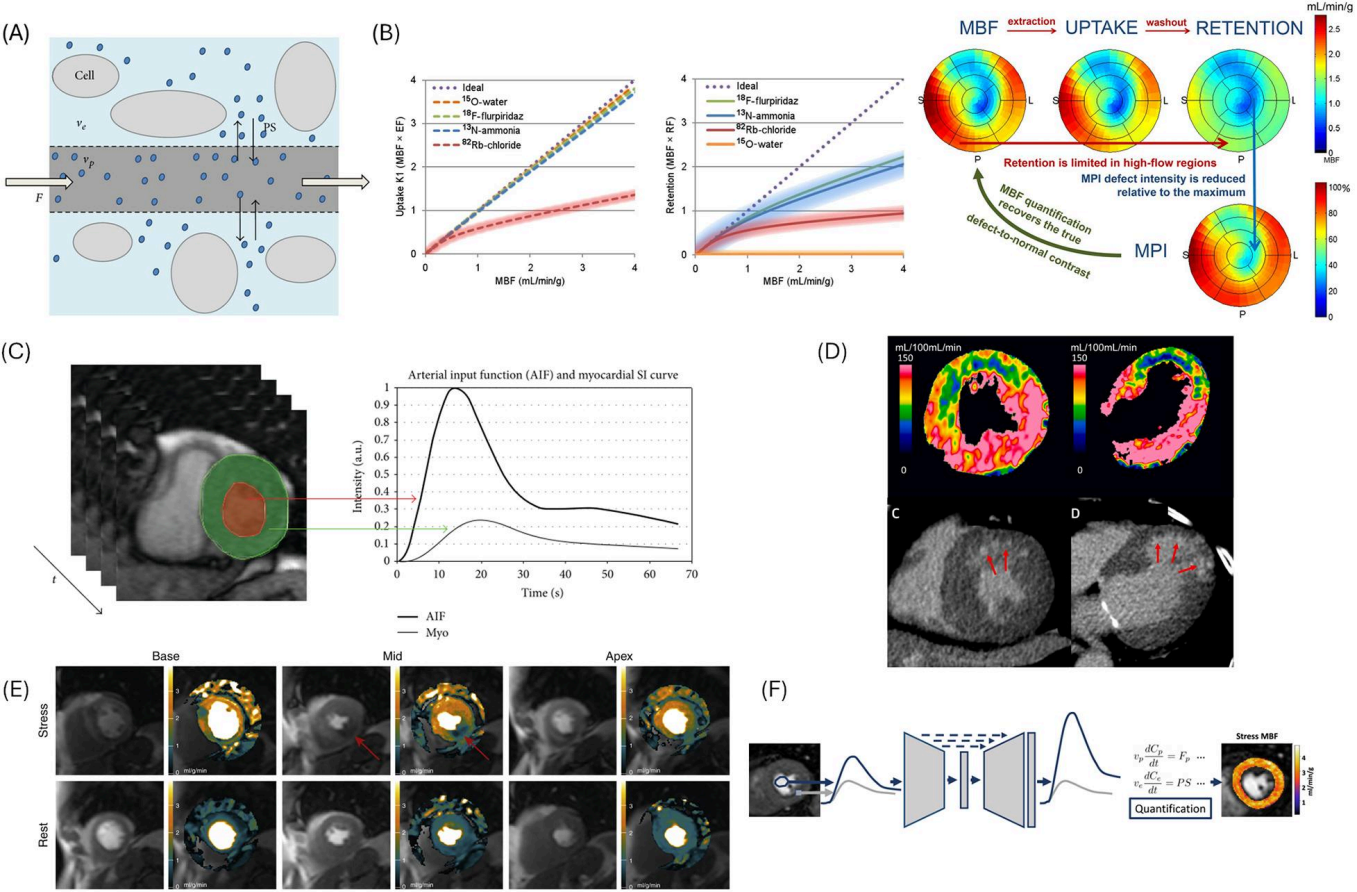
Myocardial perfusion imaging across modalities. **(A)** Compartment model. Illustration of contrast agent (blue dots) distribution in the tissue: *v*_p_ is intravascular plasma volume; *v*_e_ is the extravascular extracellular space; *F* is perfusion flow; *PS* is permeability-surface exchange rate between *v*_p_ and *v*_e_. Another parameter, the extraction fraction (*E*) denotes the proportion of contrast agent exchanged to the extravascular extracellular space. **(B)** PET tracer kinetics. Relationships between MBF and tracer uptake rate K_1_ (left), and with simplified retention modeling of tracer net uptake (middle). Shaded regions represent variability in reported values. As blood flow increases, the myocardial extraction of ^82^Rb levels off, resulting ^82^Rb uptake K_1_ reaches a plateau. In tracers such as [^15^O]H_2_O, [^13^N]NH_3_, and [^18^F]Flurpiridaz, extraction fraction keeps close to 1.0, resulting K_1_ increases proportionally to MBF. Polar maps (right) illustrate MBF, uptake, and retention along with their relationship to traditional relative MPI in example using [^13^N]NH_3_. Limited extraction fraction at higher MBF reduces uptake and retention in high-flow regions. Consequently, contrast between high- and low-MBF regions diminishes on retention images, which is made into relative perfusion images. MBF quantification restores physiologic contrast and provides an absolute scale (mL/min/g). **(C)** Time-signal intensity curve on perfusion CMR. Myocardial (green voxels) and arterial input function (red voxels) regions are sampled over time to produce time-signal intensity curves. **(D)** Representative MBF map from dynamic CTP and CT delayed enhancement. Example of a patient with prior myocardial infarction and LAD stent placement. Dynamic CTP (upper panels; left: short axis, right: long axis) demonstrates reduced MBF in the LAD territory (absolute MBF: 41 mL/100 mL/min; relative MBF: 0.25). CT delayed enhancement (lower panels; left: short axis, right: long axis) reveals subendocardial infarction in the anterior wall corresponding to the reduced MBF (arrows). **(E)** Representative automated pixel-wise MBF map by CMR. A perfusion defect in the inferior wall caused by a right coronary artery stenosis is identified (arrows). **(F)** AIF-independent perfusion CMR. Illustration of an AI-based AIF prediction model: the saturated AIF obtained from a standard acquisition (left, sampled from a round ROI in the LV blood pool) is transformed into an unsaturated AIF prediction. Combined with the myocardial tissue curve (square ROI), the inferred AIF enables quantitative stress MBF mapping without additional inputs such as dual-sequence or dual-bolus acquisition. PET, positron emission tomography; MBF, myocardial blood flow; MPI, myocardial perfusion imaging; CMR, cardiac magnetic resonance; CTP, computed tomography perfusion; LAD, left anterior descending; CT, computed tomography; AIF, arterial input function; ROI, region of interest. LV = left ventricle. Panels A and C: Adapted from Pelgrim et al. (9) (CC BY 4.0). Panel B: Adapted from Murthy et al. (12), © SNMMI. Panel D: Adapted from Kitagawa et al. (52), *J Am Coll Cardiol,* 2021, with permission from Elsevier. Panel E: Adapted from Hsu et al. (36) (CC BY 4.0). Panel F: Adapted from Scannell et al. (61) (CC BY 4.0).

Compartment modeling provides the foundation for quantitative MPI across modalities (8, 9). Understanding tracer or contrast kinetics and modeling strategies is crucial for quantitative analysis, inter-modality comparison, and physiological interpretation (10). Compartment models describe tracer exchange between intravascular and extravascular spaces, with or without a separate intracellular compartment. After delivery by blood flow, tracers cross the capillary wall into the tissue by passive diffusion or active uptake, and may return to blood or be transiently retained in myocardium; some undergo intracellular trapping via binding or metabolism (Figure 1A) (11).

The two-compartment model is most physiologically relevant. K_1_ (mL/g/min) represents the unidirectional transfer from blood to myocardium, derived from dynamic time-activity curves (TACs), time-signal intensity curves, or time-attenuation curves (TACs) in the PET/SPECT, CMR, and CT, respectively. K_1_ reflects MBF (delivery term) and the extraction fraction (a dimensionless value between 0 and 1, reflecting fraction crossing the capillary membrane), and influenced by factors such as capillary permeability—surface area product, tracer or contrast properties (molecular size, lipophilicity, protein binding, partition coefficient), tissue characteristics (fibrosis, edema), and physiological conditions (hematocrit, oxygen demand, vasodilator stress agents). K_2_ (mL/g/min) represents washout, determining the retention fraction. In a two-tissue model, K_3_ (min^−1^) represents intracellular trapping or metabolic conversion (Figure 1B) (12–14).

The arterial input function (AIF), typically obtained from the aorta or left ventricular (LV) cavity, characterizes the time-varying tracer or contrast concentration delivered to myocardium (Figure 1C). In PET, the injected dose and the first-pass extraction fraction are well characterized, often eliminating the need for explicit AIF measurement, though early studies used arterial blood sampling (11, 12). In CMR and CT, absolute MBF depends on deconvolution or compartment (tracer-kinetic) modeling. Deconvolution uses the shape of the AIF or tissue curves and not their absolute concentrations. During the time-signal intensity curve or TAC is deconvoluted with the AIF, the absolute concentration largely cancels out, isolating flow-dependent kinetics as the primary determinant of MBF (14). Compartment (tracer-kinetic) models need AIF as the input for modeling contrast exchange between compartments (9, 13). The absolute contrast concentration is directly used to fit the model and estimate K_1_ and K_2_, providing physiologically meaningful parameters. Myocardial perfusion reserve (MPR), also referred to myocardial flow reserve (MFR), represents the ratio of stress to rest MBF. Importantly, MBF values are not interchangeable across modalities due to differences in extraction fraction, retention characteristics, and modeling assumptions (6, 7, 9, 12, 15). Modality-specific implementations are described in the following sections and summarized in Table 1.

## 3 Pharmacological stress agents

Modern MPI primarily uses pharmacological stress agents. Caffeine, a methylxanthine and competitive adenosine receptor antagonist, should be avoided at least 12 h before vasodilator administration (12). Common vasodilators include non-selective adenosine receptor agonists [adenosine, adenosine triphosphate (ATP), or dipyridamole] and the selective adenosine A_2_A receptor agonist (regadenoson). Their plasma half-lives are <10 s (adenosine and ATP), 30–60 min (dipyridamole), and triphasic for regadenoson (2–4 min for initial distribution, ∼30 min for intermediate, and ∼2 h for terminal phase) (16). When stress precedes rest imaging, a sufficient time (≥ 10 min) should be allowed for the patient return to baseline. Physiological responses (e.g., heart rate, blood pressure, or symptoms) should be monitored, and aminophylline (a non-selective adenosine receptor antagonist) is used in persistent or severe adverse effects such as prolonged atrioventricular block, bronchospasm, and severe hypotension (7, 17).

## 4 Stress perfusion by PET/SPECT

### 4.1 Image acquisition protocol and tracer characteristics

PET enables MBF measurement with superior spatial and contrast resolution, lower radiation, shorter procedural time, and higher diagnostic performance than SPECT (6, 18, 19). Dynamic rest-stress imaging is standard (6, 12). CZT-SPECT MBF quantification remains challenging due to non-linear myocardial tracer uptake, but K_1_ estimation has been attempted (19). Detailed protocols are provided in the 2018 joint position paper of the Society of Nuclear Medicine and Molecular Imaging (SNMMI) and the American Society of Nuclear Cardiology (ASNC) (12), 2018 ASNC guidelines (20), and 2025 European Association of Cardiovascular Imaging (EACVI) consensus statement (21).

Common PET tracers include ^82^Rubidium (^82^Rb) and ^13^N-ammonia ([^13^N]NH_3_), with ^15^O-water ([^15^O]H_2_O) and ^18^F-Flurpiridaz ([^18^F]Flurpiridaz) mainly used in research (6, 11). Short half-life tracers (^82^Rb: 76 s; [^15^O]H_2_O: 2.03 min) require on-site cyclotrons while enable fast stress and rest imaging with lower radiation dose. Longer half-life tracers ([^13^N]NH_3_: ∼10 min; [^18^F]Flurpiridaz: ∼110 min) allow exercise stress. The representative SPECT tracers are ^201^Tl (half-life: 73 h) and ^99m^Tc-labeled compounds such as sestamibi or tetrofosmin (half-life: 6 h). ^201^Tl and ^82^Rb are potassium analogs transported into myocytes via Na^+^/K^+^ pumps. Uptake of ^99m^Tc-sestamibi, ^99m^Tc-tetrofosmin, and [^18^F]Flurpiridaz in the myocardium depends on intact mitochondria. [^13^N]NH_3_ undergoes metabolic trapping, while [^15^O]H_2_O is metabolically inert and freely diffusible (11).

### 4.2 Assessment

Visual or semi-quantitative analysis, such as summed stress and rest scores (22) remain standard but may miss balanced or diffuse ischemia (12), whereas quantitative assessment provides a more complete evaluation of myocardial perfusion.

The radioactivity concentration (counts or kBq/mL) and the resulting shape of the TACs are determined by the tracer kinetics. Absolute MBF in PET is derived as K_1_ = MBF×extraction fraction. However, the non-linearity with different degrees is noted between K_1_ and MBF in high MBF ranges among tracers (Figure 1B). Freely diffusible tracers such as [^15^O]H_2_O can be modeled with a single compartment because they rapidly equilibrate between intra- and extravascular spaces, and their washout constant (K_2_) uniquely reflects MBF (6, 12). The cut-offs or reference values are heterogenous among literatures and by the tracers [Cut-offs: 1.7–2.5 mL/min/g for MBF; for MPR, threshold of 2.0 or 2.5 is often used (1.7–2.7 in range)] (6, 12). Reporting standards are outlined in the ASNC information statement (18).

### 4.3 Performance and prognostic value of stress PET and SPECT MPI

PET by qualitative and quantitative studies shows excellent diagnostic accuracy for detecting CAD, with pooled per-patient sensitivity and specificity of 84%–90% and 81%–88%, respectively (6). In a meta-analysis of 2048 patients across 37 studies (Takx et al., 2015), quantitative PET([^15^O]H_2_O MBF-PET), as well as MRI and CT including quantitative analysis, outperformed visual SPECT or echocardiography in ruling out FFR-defined significant CAD (patient-level sensitivity/specificity: PET 84/87%, MRI 89/87%, CT 88/80%, SPECT 74/79%, echocardiography 69/84%) (23). Quantitative PET exceeds qualitative relative perfusion, regardless of tracer type (6). In [^13^N]NH_3_ PET, absolute stress MBF outperformed relative uptake for detecting ≥70% stenosis by ICA (accuracy 84% vs. 72%, *p* < 0.01) (24).

For prognostication in suspected or known CAD, impaired hyperemic MBF by [^15^O]H_2_O PET (<2.65 mL/min/g globally or <2.10 mL/min/g regionally) independently predicted death or myocardial infarction, after adjustment for clinical characteristics and MFR (global: HR = 2.55, *p* = 0.008; regional: HR = 2.13, *p* = 0.04) (Bom et al., 2020) (25). In another study, only MFR independently predicted MACE, outperforming absolute MBF (Benz et al., 2021) (26). Because MFR incorporates resting perfusion, it reflects broader physiological influences (e.g., hypertension, age) and offers a more comprehensive risk profile than MBF (6). Supporting this, a systematic review (Juárez-Orozco et al., 2018) of 6804 patients across 8 studies (^82^Rb, [^13^N]NH_3_, [^15^O]H_2_O) confirmed stronger prognostic value for MFR over stress MBF in predicting MACE and cardiac mortality (27). Longitudinal quantitative PET increasingly informs clinical decision-making (28, 29).

In head-to-head comparison with [^15^O]H_2_O PET, quantitative CZT-SPECT overestimated MBF, but MFR was comparable (30). In 303 CAD patients, including 100 INOCA (Li et al., 2023), SPECT-MFR <2.0 predicted higher MACE (log-rank *P* = 0.0019), with each 1-unit increase reducing risk by ∼66% in INOCA and ∼64% overall (31). A meta-analysis (Baksa et al., 2025) confirmed diagnostic and prognostic value of CZT-SPECT MFR in suspected or known CAD, with pooled sensitivity 78.5% and specificity 89.3% vs. PET, and impaired SPECT-MFR independently predicting MACE (32).

## 5 Stress perfusion by CMR

### 5.1 Image acquisition protocol

The 2025 Society for Cardiovascular Magnetic Resonance (SCMR) Expert Consensus Statement recommends a stress-rest protocol with adenosine as the preferred vasodilator. Free-breathing sequences with motion correction are preferred, though breath-hold acquisitions remains acceptable (7).

AIF and high-resolution myocardial perfusion images can be obtained using dual bolus (DB) or dual sequence (DS). DB can be performed on any scanner but requires a diluted (10%; ∼0.005–0.01 mmol/kg) bolus followed by full-dose injection (0.05–0.1 mmol/kg). DS uses a single bolus with a low-resolution spoiled gradient echo with short-saturation preparation time (for AIF) followed by high-resolution perfusion imaging; it is easier to implement (33). In DS acquisition, high contrast concentration in the blood pool can cause signal deviation from linearity due to T1 and T2* saturation effects, whereas in DB, the low-dose bolus for AIF keeps blood signal nonlinearity negligible in practice (7, 34, 35). Post-processing includes motion correction, signal non-linearity correction, baseline correction, coil sensitivity correction, and spatial filtering (7, 36). Detailed protocols are summarized in the 2025 SCMR Statement (7).

### 5.2 Assessment

Visual interpretation forms the basis of assessment, with limitations from inter-observer variability and dark-rim artifacts (37). Semi-quantitative “MPR” analysis uses the upslope or area under the myocardial time-signal intensity curve normalized to the LV blood pool (i.e., AIF); a stress/rest ratio >2 is generally considered normal (7). However, results depend on hemodynamics, imaging parameters, and contrast kinetics (13).

Quantitative perfusion CMR provides pixel-wise absolute MBF (mL/min/g) and MPR (i.e., MFR) with high spatial resolution (7). Although perfusion quantification is based on two compartment modeling (13), the high extraction fraction (effectively near 1 under clinical doses in CMR perfusion) and rapid gadolinium exchange allow practical use of single-compartment deconvolution approaches (10, 13, 33) (e.g., Fermi function/model-constrained deconvolution). Prior to deconvolution, DS requires AIF saturation correction and signal intensity-to-concentration conversion for both the AIF and myocardium, derived from two different sequences (7, 34, 35). In DB, this conversion can be bypassed, but scaling of the AIF by the dose ratio is required (13). Tracer-kinetic (compartmental) estimates the analogous to K_1_ by clearer physiological interpretation (8, 9). Conversion from signal intensity to concentration may be optional, as signal nonlinearities can be incorporated into the model. Perfusion defects are recommended to be reported as percentage of total myocardium (7).

### 5.3 Performance and prognostic value of stress CMR

Stress CMR demonstrates excellent diagnostic accuracy for hemodynamically significant CAD (38). In the CE-MARC study (2012) which visually scored the ischemic hypoperfusion (39), multiparametric CMR outperformed SPECT with higher sensitivity (86.5% vs. 66.5%) and similar specificity (83.4% vs. 82.6%). Fully automated pixel-wise MBF on DS imaging (Hsu et al., 2018) reported per-patient sensitivity and specificity of 82.9% and 80.0%, respectively, for detecting significant CAD by ICA. The stress and rest MBF from ischemic area was 0.92 ± 0.36 and 0.74 ± 0.25 mL/g/min, respectively, while 3.67 ± 0.79 and 1.22 ± 0.33 mL/g/min, respectively, in healthy volunteers (36).

For prognosis, MR-INFORM (2019) which followed 918 patients with stable angina for a median of 1 year, demonstrated less revascularization (35.7% vs. 45.0%, *P* = 0.005) and non-inferiority for major outcomes at 1 year (3.6% vs. 3.7%) in visually semi-quantified CMR-guided strategy to FFR-guided management. Freedom from angina at 12 months was also similar (49.2% vs. 43.8%, *p* = 0.21) (40). In a study using AI-derived MBF maps (Knott et al., 2020) on 1049 patients with suspected and known CAD, each 1 mL/g/min decrease in stress MBF predicted death (HR =1.93, *P* = 0.028) and MACE (HR = 2.14, *P* < 0.0001). In a sub-group suspected for microvascular disease, MPR remained independently associated with death and MACE, with stress MBF remaining associated with MACE only (41).

## 6 Stress perfusion by cardiac CT

### 6.1 Image acquisition protocol

The Society of Cardiovascular Computed Tomography (SCCT) expert consensus recommends adding CT perfusion (CTP) for patients at high risk of obstructive CAD, including prior interventions or significant calcification, or stenoses of indeterminate functional significance (42). The choice of rest-first vs. stress-first depends on clinical context: patients with known CAD or extensive coronary calcium may undergo stress first, whereas coronary CTA is prioritized when anatomic information is lacking (42). Two primary approaches are static and dynamic CTP. Static CTP captures a single phase during contrast injection, enabling qualitative or semi-quantitative assessment; coronary CTA also serves as a resting static CTP. Reported radiation doses on 320-row scanners range from 2.5 to 9.3 mSv (42). Dynamic CTP acquires serial images during first-pass contrast to generate TACs proportional to MBF, with higher radiation exposure (5.3 to 13.1mSv) (42). Dual-energy CTP improves iodine visualization and mitigates beam-hardening and low contrast-to-noise ratio (CNR) issues (43), with reported doses of 4.2 to 16.5mSv (42). Dynamic CTP requires myocardial coverage (7–10 cm) during a 20–30 s breath-hold with high temporal resolution; shuttle mode is used in scanners with limited z-axis coverage, acquiring CT data at two alternate table positions by moving the table back and forth (42, 44). Detailed protocols are available in the 2020 SCCT expert consensus (42) and in publications (44, 45).

### 6.2 Assessment

In static CTP, absolute MBF is not assessable and perfusion defects are typically reported by the summed stress score (SSS) (42, 46). Dynamic CTP quantifies MBF typically using single-compartment indicator-dilution modeling and deconvolution (47), supported by the linear relationship between Hounsfield units (HU) and iodine concentration (48) (Figure 1D). Patlak tracer-kinetic approach estimates K_1_ via linear least-squares fitting without full multi-compartment modeling, simplifying the analytical procedures (14). Dynamic CTP also enables semi-quantitative TAC analysis normalized to AIF, with ischemic regions showing lower upslope and peak (49). CAD-RADS 2.0 CT reporting system incorporates ischemia detection as *modifier “I”*, with three severity categories of I+, I-, and I ±  (50).

### 6.3 Performance and prognostic value of stress CTP

Static stress CTP has been validated across multiple reference standards. In CORE320 (2014), static stress CTP plus coronary CTA was compared with stress SPECT in 381 patients with suspected or known CAD. Using ≥50% stenosis on ICA as reference, per-patient sensitivity and specificity were 88% and 55% for CTP vs. 62% and 67% for SPECT, with higher sensitivity in CTP for left main and multivessel disease (46). A 2019 meta-analysis of 5,330 patients from 54 studies comparing anatomical and functional CT for detecting FFR-defined significant CAD demonstrated that CTP, from both dynamic and static studies, improved per-patient diagnostic accuracy over CTA (sensitivity and specificity: 83% and 79% for CTP; 94% and 48% for CTA). At the vessel level, dynamic CTP showed higher sensitivity (85% vs. 72%) but lower specificity (81% vs. 90%) than static CTP (51).

A multicenter dual-source dynamic stress CTP study (2021) utilized optimal cutoffs of 116 mL/100 mL/min (absolute MBF) and 0.71 (relative MBF) for detecting invasive FFR-defined significant stenoses. Dynamic CTP added incremental value over CTA, improving per-patient accuracy (from 64% to 74%, *p* < 0.001) and specificity (from 36% to 75%, *p* < 0.001), though with reduced sensitivity (from 93% to 72%, *p* < 0.001) (52). A 2024 meta-analysis of 2190 patients from 23 studies reported pooled MBF of 0.92 vs. 1.39 mL/min/g in ischemic vs. non-ischemic regions (*P* < 0.001). For detecting FFR-defined significant stenosis, patient-based pooled AUC was 0.92 with sensitivity 0.82 and specificity 0.86. CT-MBF also predicted adverse events (pooled HR =4.98) (53).

Dynamic CTP also informs clinical decision making and prognostication. A 2020 study in patients with suspected CAD showed that a dynamic CTP-guided strategy significantly reduced angiography without revascularization compared with a CTA-only strategy (10.8% vs. 50.0%, *p* < 0.0001) without increasing 1-year MACE (54). In a 2025 study of 226 patients followed for a median of 3.4 years, the relative MBF ratio (r-MBF), defined as the ratio of lowest to highest MBF regions, was the only independent predictor of MACE (HR = 0.82, *p* = 0.01), whereas CT-FFR was not (55).

## 7 Discussion: future perspectives

Standardization of acquisition and analysis protocols is needed to mitigate variability in MBF within and across modalities, which are not yet interchangeable (6, 7, 9, 12, 15). Pixel-wise MBF mapping should become the dominant approach for future perfusion studies.

Advances in acquisition protocols, including DS acquisition in CMR, may improve accuracy and reproducibility. A notable topic not covered in earlier sections is non-contrast perfusion CMR based on native T1 mapping; it may expand access for patients with renal dysfunction and those requiring long-term follow-up (56, 57). CT perfusion is also progressing toward more quantitative perfusion assessment with lower contrast and radiation doses (42). Iodine mapping in spectral CTs such as dual-energy and photon-counting CT shows promise for future MBF quantification.

The wider adoption of AI-assisted workflows, including automated preprocessing, AIF detection and modeling (58), MBF map generation (36, 41), and prognostication (59), will further streamline analysis and reduce operator dependence (Figure 1E). Deep neural networks trained on large datasets can infer AIF from standard high-resolution perfusion images and may enable AIF-independent quantification in CMR and CT (Figure 1F) (60, 61).

## 8 Conclusion

Myocardial perfusion reflects both macrovascular and microvascular coronary flow, which deserves reappraisal with modern PET, CMR, and CT techniques enabling absolute MBF with improved diagnostic and prognostic performance. Standardized acquisition and analysis protocols are warranted to mitigate variability in MBF measurements across modalities. AI-assisted workflows will further support broader clinical adoption of MPI.
